# Minimising multi-centre radiomics variability through image normalisation: a pilot study

**DOI:** 10.1038/s41598-022-16375-0

**Published:** 2022-07-22

**Authors:** Víctor M. Campello, Carlos Martín-Isla, Cristian Izquierdo, Andrea Guala, José F. Rodríguez Palomares, David Viladés, Martín L. Descalzo, Mahir Karakas, Ersin Çavuş, Zahra Raisi-Estabragh, Steffen E. Petersen, Sergio Escalera, Santi Seguí, Karim Lekadir

**Affiliations:** 1 Artificial Intelligence in Medicine Lab (BCN-AIM), Barcelona, Spain; 2 Vall d’Hebron Institut de Recerca (VHIR), Barcelona, Spain; 3 CIBER-CV, Instituto de Salud Carlos III, Madrid, Spain; 4 Department of Cardiology, Hospital Universitari Vall d’Hebron, Barcelona, Spain; 5 Department of Medicine, Universitat Autònoma de Barcelona, Bellaterra, Spain; 6 Cardiac Imaging Unit, Cardiology Service, Hospital de la Santa Creu i Sant Pau, Universitat Autonoma de Barcelona, Barcelona, Spain; 7 Department of Cardiology, University Heart and Vascular Center Hamburg, Hamburg, Germany; 8 DZHK (German Center for Cardiovascular Research), Hamburg, Germany; 9 William Harvey Research Institute, NIHR Barts Biomedical Research Centre, Queen Mary University London, London, UK; 10 Barts Heart Centre, St Bartholomew’s Hospital, Barts Health NHS Trust, London, UK; 11 Health Data Research UK, London, UK; 12 Alan Turing Institute, London, UK; 13 Computer Vision Center, Universitat Autonoma de Barcelona, Barcelona, Spain; 14 Department of Intensive Care Medicine, University Medical Center, Hamburg Eppendorf, Hamburg, Germany

**Keywords:** Diagnostic markers, Cardiovascular diseases, Computer science

## Abstract

Radiomics is an emerging technique for the quantification of imaging data that has recently shown great promise for deeper phenotyping of cardiovascular disease. Thus far, the technique has been mostly applied in single-centre studies. However, one of the main difficulties in multi-centre imaging studies is the inherent variability of image characteristics due to centre differences. In this paper, a comprehensive analysis of radiomics variability under several image- and feature-based normalisation techniques was conducted using a multi-centre cardiovascular magnetic resonance dataset. 218 subjects divided into healthy (n = 112) and hypertrophic cardiomyopathy (n = 106, HCM) groups from five different centres were considered. First and second order texture radiomic features were extracted from three regions of interest, namely the left and right ventricular cavities and the left ventricular myocardium. Two methods were used to assess features’ variability. First, feature distributions were compared across centres to obtain a distribution similarity index. Second, two classification tasks were proposed to assess: (1) the amount of centre-related information encoded in normalised features (centre identification) and (2) the generalisation ability for a classification model when trained on these features (healthy versus HCM classification). The results showed that the feature-based harmonisation technique ComBat is able to remove the variability introduced by centre information from radiomic features, at the expense of slightly degrading classification performance. Piecewise linear histogram matching normalisation gave features with greater generalisation ability for classification ( balanced accuracy in between 0.78 ± 0.08 and 0.79 ± 0.09). Models trained with features from images without normalisation showed the worst performance overall ( balanced accuracy in between 0.45 ± 0.28 and 0.60 ± 0.22). In conclusion, centre-related information removal did not imply good generalisation ability for classification.

## Introduction

For the last decade, there has been a great amount of research devoted to identifying and improving quantitative image biomarkers for precise diagnosis, risk assessment and patient stratification for different pathologies. In particular, radiomics seems to be a promising technique to quantify image-derived biomarkers based on shape, intensity and higher-order texture patterns for a region of interest defined a priori, since it is able to characterise image patterns that are hardly visible to the naked eye.

These computer-extracted features have the potential to perform an exhaustive analysis of medical images as shown in the literature, predominantly in oncology^1^ but also more recently for neurodevelopmental disorders^2^ or cardiovascular disease^3^. However, radiomic features have proven to be highly sensitive to changes in scanning protocols and scanner manufacturers, resulting in a limited reproducibility^4,5^ (see also the exhaustive reviews by Yip and Aerts^6^ and by Traverso et al.^7^) and thus posing an important problem that needs to be solved before implementing these techniques in clinical practice. Despite this, the majority of previous research considered single-institution datasets, due in part to the difficulty in obtaining imaging studies from multiple centres. More recently, several works using multi-centre studies have assessed the robustness of this technique (see for example Raisi-Estabragh et al.^8^, for a test-retest study). A number of works have proposed harmonisation guidelines for computed tomography (CT) or positron emission tomography in multi-centre scenarios, while no guideline is available for magnetic resonance imaging (MRI), where the lack of a standard intensity grayscale—such as Hounsfield units in CT—poses further difficulty (see Da Ano et al.^9^ and references therein).

All previous multi-centre MRI radiomics studies focused either on brain or cancer imaging. Due to the lack of multi-centre cardiac imaging radiomics literature, a detailed introduction about brain and cancer imaging is presented. Two types of techniques are used to standardise features across institutions: image- and feature-based transformations.

At the image level, the most common techniques are image intensity normalisation (mean subtraction and division by the standard deviation) or image intensity rescaling to a fixed range (usually from 0 to 1). Other more sophisticated techniques exist, such as bias field correction, isotropic resampling, histogram matching and piecewise linear histogram matching (PLHM). Finally, some techniques are inherently defined for brain imaging and were not considered in this study. Um et al. used T1-weighted MRI brain scans to assess radiomics variability across two different institutions after five image preprocessing techniques were applied, including global and region of interest (ROI) rescaling, bias correction, isotropic resampling and histogram matching^5^. They concluded that histogram matching is the best technique for reducing feature variability and successfully discriminate between different patient subgroups with glioblastoma. Isaksson et al. evaluated the effect of four normalisation techniques on classification performance to identify prostate cancer in T2-weighted MRI^10^. The normalisation method that resulted in the best classification accuracy was the PLHM transformation using intensities from healthy prostate as reference instead of the whole image to extract landmarks. Finally, Carré et al. standardised brain MRI studies using three different intensity normalisation techniques in order to find their effects on radiomics robustness, being image intensity normalisation the technique that yielded the best results^11^.

At the feature level, Chatterjee et al. improved the robustness of radiomics from images of primary uterine adenocarcinoma by applying feature normalisation for each institution dataset independently^12^. Orlhac et al., instead, used the empirical Bayes harmonisation method—also referred to as ComBat^13^—to remove inter-centre variability^14^. The transformed features resulted in a sensitivity increase for distinguishing between Gleason grades in prostate cancer studies and in similar distributions for features from brain scans for 1.5T and 3T machines.

In this work, a multi-centre cardiac MRI dataset was considered to analyse the effect of several image- and feature-based normalisation techniques over radiomic features variability and model generalisation across institutions.

## Results

### Feature variability

**Figure 1:**
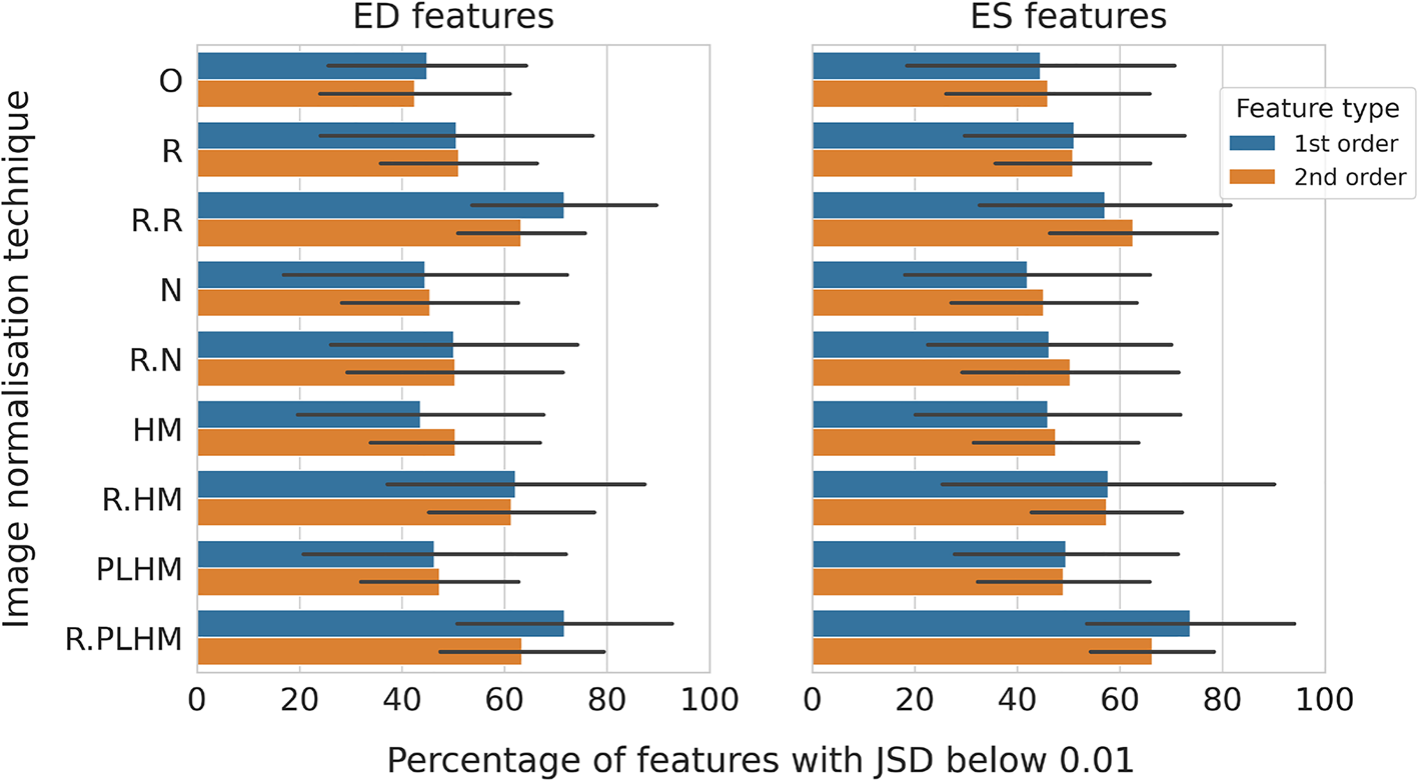
Percentage of first and second order features below the 0.01 JSD threshold for healthy subjects. Results are averaged over centre pairs and ROI and presented separately for ED and ES frames. Only features with square cross-correlation below 0.9 were considered. The black lines represent the standard deviation. *O* original images (without normalisation), *R* image intensity rescaling, *N* image intensity normalisation, *HM* histogram matching and *PLHM* piecewise linear histogram matching. An “R.” in front of a method means that it is applied at ROI level.

Texture features variability showed a great disparity depending on the preprocessing method under consideration (see Fig. 1). For both the end-diastole (ED) and the end-systole (ES) frames, the percentage of features with similar distributions across institutions (Jensen–Shannon divergence, JSD, below 0.01) was obtained after the removal of highly correlated features ($R^2 \ge 0.9$). After this step, the amount of first/second order ED (ES) features remaining were 7/38 (8/47) for LV, 9/48 (9/49) for MYO and 9/42 (8/47) for RV (the square correlation heatmaps are shown in Fig. S2 in the supplementary material). The highest percentage of features with similar distributions across institutions was obtained when applying ROI-based PLHM or ROI-based rescaling as shown in Fig. 1 (with a maximum of 74% for first order features and of 66% for second order features). More specifically, these two methods showed a significant difference in distribution similarity for first order features when compared to other methods, and only ROI-based histogram matching showed comparable results for second order ED features (all tests with p-values below 0.01, Mann–Whitney *U* test).

In contrast, the proportion of features below the 0.01 JSD threshold was the lowest for the methods applied at the whole image level, except for rescaling, and for images without any normalisation (N, HM, PLHM and O in Fig. 1). No significant difference was found between these normalisation methods and original images for both ED and ES features (p-values greater than 0.01, Kruskal–Wallis test). The proportion of features below the given threshold was reduced to less than 51%, indicating that feature distributions were less similar for these methods. Additionally, the large standard deviation, represented by the black horizontal bars in Fig. 1, was associated to differences depending on the ROIs and, especially, on the centre pairs being compared (see Fig. S1 and S3 in the supplementary material for a detailed comparison of these factors).

**Figure 2:**
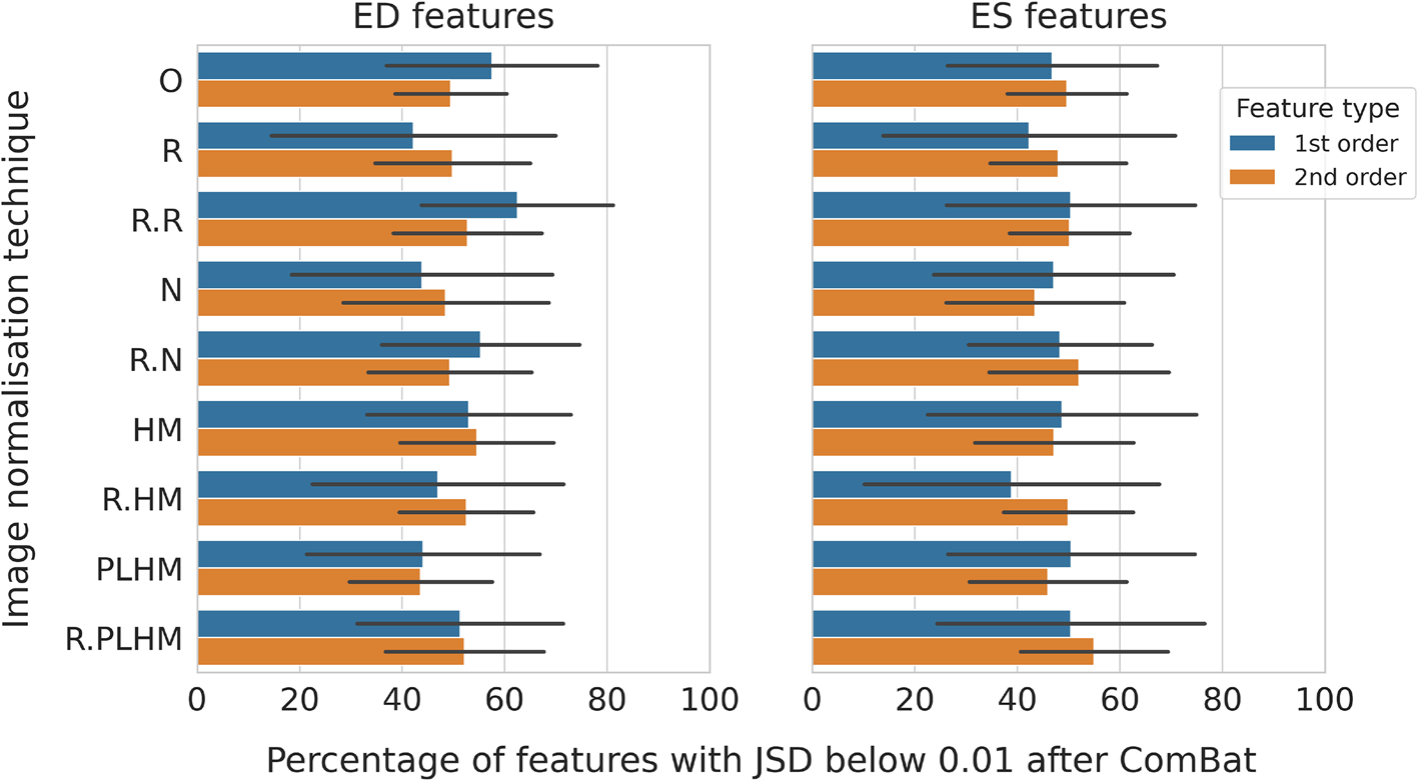
Percentage of first and second order features below the 0.01 JSD threshold for healthy subjects after the application of the feature-based harmonisation tool ComBat. Results are averaged over centre pairs and ROI and presented separately for ED and ES frames. Only features with square cross-correlation below 0.9 were considered. The black lines represent the standard deviation. *O* original images (without normalisation), *R* image intensity rescaling, *N* image intensity normalisation, *HM* histogram matching and PLHM: piecewise linear histogram matching. An “R.” in front of a method means that it is applied at ROI level.

The application of the ComBat harmonisation method had an averaging effect, reducing the proportion of features with similar distributions for the most robust methods in the previous paragraph and increasing it for the least robust methods, as shown in Fig. 2. Thus, smaller differences were found between methods after the application of ComBat. Specifically, no significant difference was found between four methods for ED features (R.R, R.N, R.PLHM and O in Fig. 2), and for three methods for ES features (R.R, R.N and R.PLHM in Fig. 2, p-values greater than 0.01, Kruskal–Wallis test).

Among all feature families, Gray Level Size Zone Matrix (GLSZM), Gray Level Run Length Matrix (GLRLM) and Gray Level Dependence Matrix (GLDM) presented the highest dissimilarities among distributions after the application of ROI-based PLHM normalisation in general, as demonstrated by the greater JSD values in Table 1. Gray Level Co-occurrence Matrix (GLCM) and first order features obtained the best similarity scores. As noted above, the JSD was averaged over all family of features to an approximate value of 0.011 after the application of ComBat. The features found with the most dissimilar distributions (standard deviation of the JSD distribution greater than 0.01) in both cardiac time frames, ED and ES, prior to the application of ComBat were zone variance, large area emphasis and large area low gray level emphasis (GLSZM), kurtosis (1st order) and gray level non-uniformity (GLDM). Some examples of the effects of ComBat and PLHM over the different distributions per centre are presented in Fig. S4 in the supplementary material.

**Table 1:** Mean and standard deviation (in parenthesis) for JSD for distributions of features obtained after the application of R.PLHM normalisation on healthy patients. Results are presented separately for ED and ES frames and for each feature family before and after the application of ComBat harmonisation. Only features with square cross-correlation below 0.9 were considered. Values are averaged over ROI. Numbers in blue stand for non-significant differences in the JSD distributions when compared to first order features according to the Mann–Whitney *U* test at the 0.01 level.

Family	Without combat		With combat	
	ED	ES	ED	ES
1st order	0.009 (0.009)	0.008 (0.007)	0.012 (0.011)	0.011 (0.008)
GLCM	0.008 (0.007)	0.009 (0.009)	0.013 (0.012)	0.012 (0.013)
GLDM	0.011 (0.010)	0.010 (0.008)	0.013 (0.013)	0.011 (0.010)
GLRLM	0.012 (0.011)	0.010 (0.007)	0.011 (0.010)	0.011 (0.009)
GLSZM	0.011 (0.011)	0.011 (0.010)	0.013 (0.012)	0.011 (0.010)

### Centre identification

**Figure 3:**
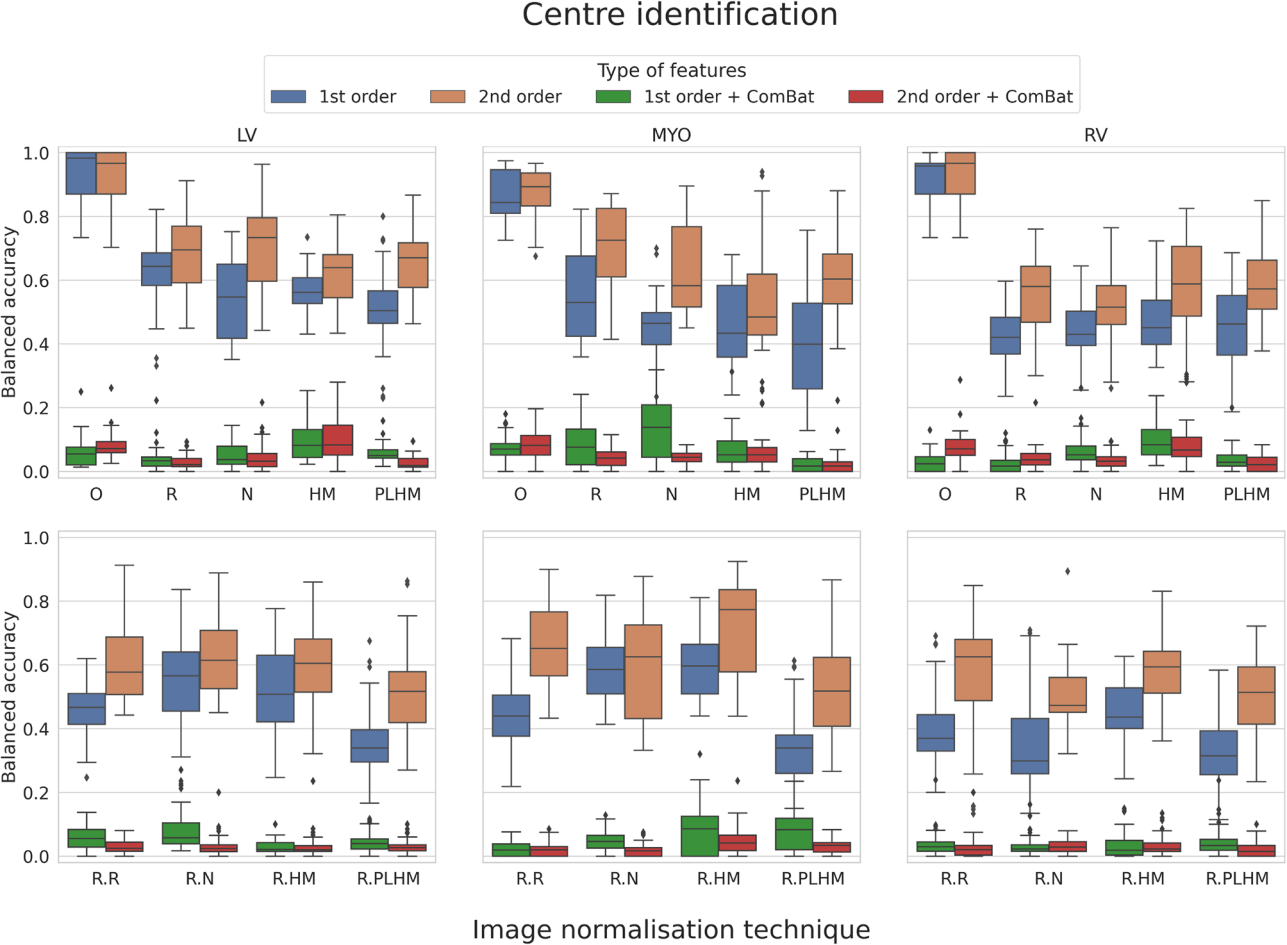
Balanced accuracy of random forest models when predicting the centre of origin of healthy subjects for first and second order texture features before and after the application of ComBat harmonisation. The row above corresponds to image preprocessing techniques applied at the whole image level, while in the row below they are applied at the ROI level. *O* original images (without normalisation), *R* image intensity rescaling, *N* image intensity normalisation, *HM* histogram matching, *PLHM* piecewise linear histogram matching. An “R.” in front of a method means that it is applied at ROI level.

When assessing the centre information encoded in the extracted features, second order texture features carried more information in general than first order features, as demonstrated by the differences in balanced accuracy for classifiers trained with healthy subjects in Fig. 3 (orange and blue boxes). Features from original images (without normalisation) were the most discriminative features with testing accuracy above 0.87 (± 0.07–0.11) for the three ROIs under consideration and for both feature types, first order and texture features. When comparing normalisation techniques at whole image level, no clear method showed a greater reduction in the centre information consistently across ROIs and feature types. (Fig. 3, orange and blue boxes in the top row).

When normalisation was applied at ROI level, larger differences appeared depending on the method and the ROI under consideration (Fig. 3, orange and blue boxes in the bottom row). Regarding methods that did not use ComBat, ROI-based PLHM consistently reduced the ability of models to infer the centre of origin for each sample for first order features extracted from LV and MYO, and for second order features from LV, achieving the lowest performance (p-values below 0.01, Mann–Whitney *U* test). For the RV, however, three methods (R.R, R.HM and R.PLHM) showed comparable accuracy (p-value greater than 0.01, Kruskal–Wallis test). Finally, ComBat harmonisation was able to remove centre information from features almost entirely for most normalisation techniques and original images, as shown by the red and green boxes in Fig. 3.

When models were trained only with HCM patients, the general behavior between methods observed for healthy subjects was reproduced, but the accuracy for identifying the centre was reduced for all methods prior to using ComBat harmonisation. See Fig. S5 in the supplementary material, for more details.

### Generalisation

**Figure 4:**
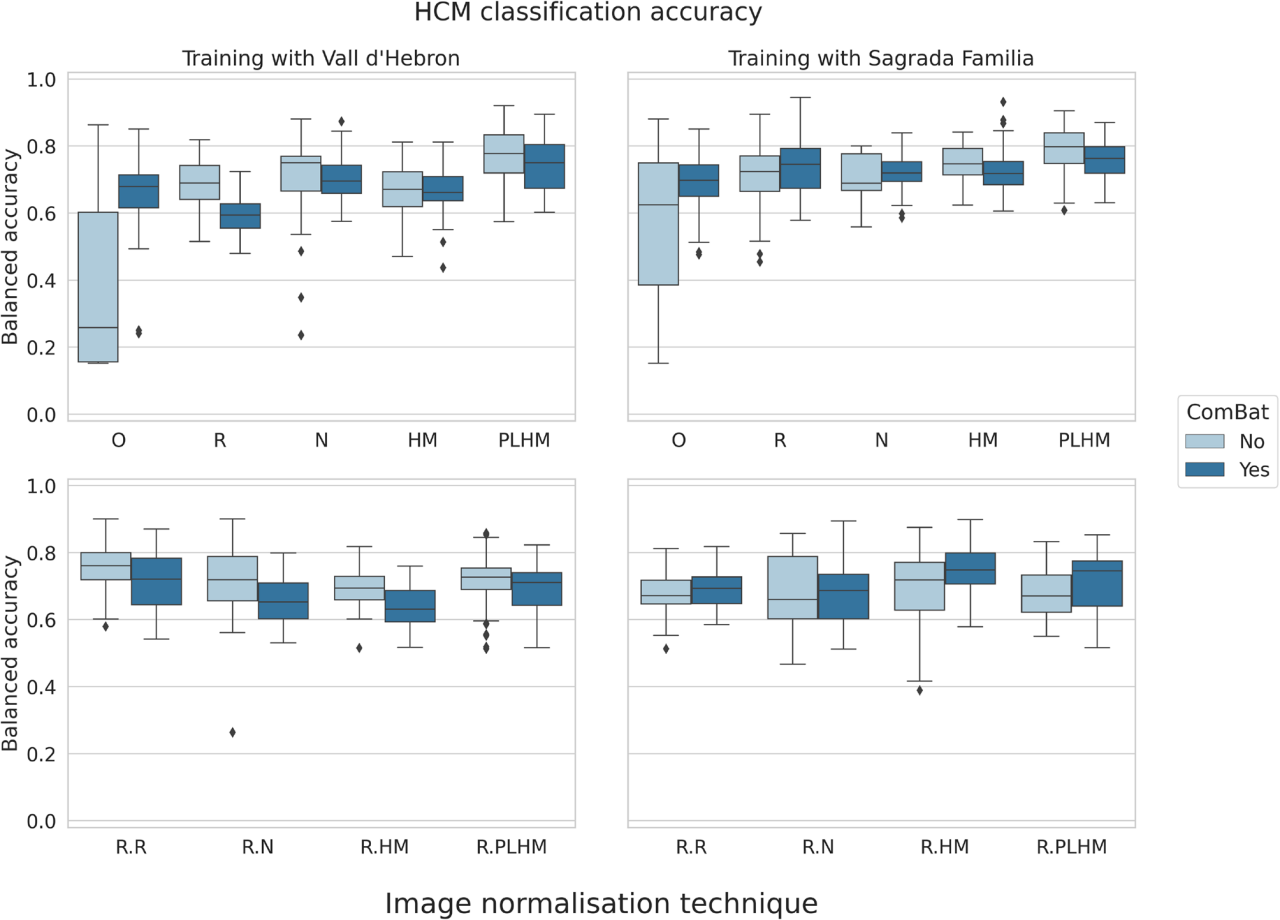
Balanced accuracy of random forest models on unseen centres for classification of HCM versus healthy patients. All models were trained with a combination of first and second order texture features from all ROIs. The first column corresponds to models trained with features extracted from Vall d’Hebron studies, while models in the second column were trained with features from Sagrada Familia studies. The row above corresponds to image preprocessing techniques applied at the whole image level, while in the row below they are applied at the ROI level. *HCM* hypertrophic cardiomyopathy, *O* original images (without normalisation), *R* image intensity rescaling, *N* image intensity normalisation, *HM* histogram matching, *PLHM* piecewise linear histogram matching. An “R.” in front of a method means that it is applied at ROI level.

With regards to the patient classification task into healthy and hypertrophic cardiomyopathy (HCM) groups in unseen centres (see (Fig. 4), models trained with features from original images (without normalisation) showed the worst performance. With regards to models trained with features from images normalised at whole image level (Fig. 4, upper row), N and PLHM methods were significantly better than other methods and performed similarly when trained with studies from Vall d’Hebron, while PLHM was significantly better when trained with studies from Sagrada Familia (all p-values below 0.01, Mann–Whitney *U* test, after the Bonferroni correction for multiple comparison).

When images underwent ROI-based normalisation, ROI-based rescaling and ROI-based normalisation performed on par and significantly better than other models when trained with Vall d’Hebron studies (p-values below 0.01, Mann–Whitney *U* test), while no method was significantly better than others when trained with studies from Sagrada Familia (p-value greater than 0.01, Kruskal–Wallis test).

The application of ComBat reduced the accuracy slightly in general, but the difference was only significant for rescaling, ROI-based normalisation and ROI-based histogram matching when training with Vall d’Hebron studies, and for whole image and ROI-based histogram matching and ROI-based PLHM when training with studies from Sagrada Familia (p-values below 0.01, Mann–Whitney *U* test, after Bonferroni correction for multiple comparison).

For both types of models, trained with Vall d’Hebron and Sagrada Familia studies, the best accuracy was obtained when using features extracted after applying the PLHM transformation and without ComBat harmonisation: $78.3\%\pm 8.4$ and $79.2\%\pm 8.8$, respectively.

In more detail, for models trained with features from Vall d’Hebron studies, the highest accuracy was 0.783 (median: 0.792 [0.745, 0.845]), obtained after PLHM without the application of ComBat. When ComBat harmonisation was used, the highest accuracy was obtained after the application of the same image normalisation technique but was reduced to 0.771 (median: 0.775 [0.694, 0.826]). For models trained with features from Sagrada Familia studies, the best accuracies were again obtained for PLHM and were 0.783 (median: 0.792 [0.728, 0.850]) and 0.762 (median: 0.762 [0.712, 0.811]) before and after the application of ComBat harmonisation, respectively. For these models, features mostly from the myocardium (MYO) were among the most important features for the model prediction according to the Gini importance^15^. The top 20 most important features contained mean and median intensity, kurtosis and skewness (1st order), joint average and autocorrelation (GLCM) and run length non-uniformity and long run high gray level emphasis (GLRLM).

**Figure 5:**
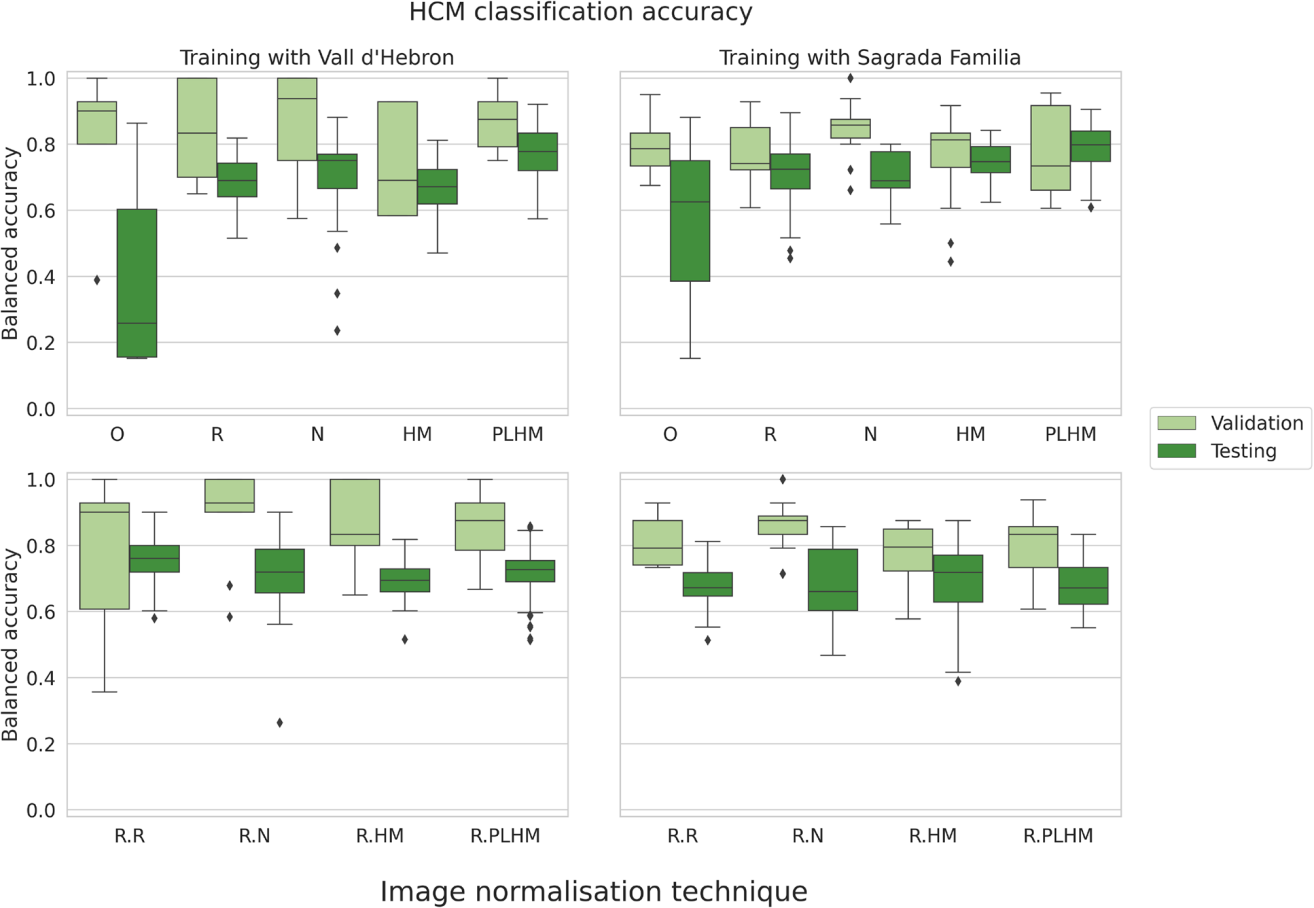
Balanced accuracy of random forest models on the validation set (same domain) versus the testing set (unseen centres) for classification of HCM versus healthy patients. Results are presented without ComBat harmonisation. All models were trained with a combination of first and second order texture features from all ROIs. The first column corresponds to models trained with features extracted from Vall d’Hebron studies, while models in the second column were trained with features from Sagrada Familia studies. The row above corresponds to image preprocessing techniques applied at the whole image level, while in the row below they are applied at the ROI level. *HCM* hypertrophic cardiomyopathy, *O* original images (without normalisation), *R* image intensity rescaling, *N* image intensity normalisation, *HM* histogram matching, *PLHM* piecewise linear histogram matching. An “R.” in front of a method means that it is applied at ROI level.

When comparing the accuracy between validation (same institution) and testing (unseen institutions) sets, models that obtained the highest accuracy on validation generalised worse to new unseen centres (Fig. 5). Importantly, models trained with features from ROI-based normalisation methods showed relatively similar generalisation performance among them, even though some suffered from overfitting. Within normalisation methods at the whole image level, features extracted after PLHM obtained the best testing accuracy despite their lower performance in validation when compared to other techniques.

## Discussion

Radiomic features are promising biomarkers for better disease characterisation. However, their variability across centres makes it difficult to establish reproducible biomarkers based on them^6^. In this study, a comprehensive analysis was carried out to assess feature variability across centres as well as model generalisation for a classification task after the application of several image normalisation techniques and a feature-based harmonisation technique (ComBat).

Based on the results presented, ROI-based PLHM is a good normalisation technique to preserve similar feature distributions across domains (see Fig. 1) and to reduce the amount of centre-related information encoded in radiomic features compared to original images (see Fig. 3). In brain MRI literature, however, the transformations that yielded less feature variability and more similar distributions were histogram matching and image intensity normalisation^5,11^, although Um et al. did not consider PLHM in their work.

At feature normalisation level, ComBat satisfactorily removed centre-related information to the point that models were not able to discriminate between features depending on the institution of origin of each scan. As a drawback, the final feature distributions at different centres were less similar than before the application of ComBat according to the JSD.

With regards to generalisation ability, models trained with features from original images resulted in poor performance for differentiation of healthy subjects from HCM patients in unseen centres, highlighting the importance of normalisation techniques for multi-centre studies. Models trained with features extracted after PLHM normalisation obtained the highest accuracy. In this method, average landmarks are obtained for a reference population, while for histogram matching the reference was only one subject. This could explain why these methods showed differences in performance despite relying on the same principle, since defining a template using only one subject could introduce unwanted bias in the analysis. Moreover, the selection of a particular population or subject as reference template in these methods may affect the results, especially for histogram matching (see Figs. S6 and S7 in the supplementary material).

Importantly, successful centre-related information removal from radiomic features does not imply greater generalisation ability. In fact, ROI-based PLHM and ComBat harmonisation methods were not among the best generalisation techniques for the HCM classification task (Fig. 4, bottom row). When compared with the brain MRI literature, Orlhac et al. did find an improvement in sensitivity for differentiating between low and high risk patient groups when using ComBat harmonisation, although the authors did not compare different image normalisation techniques^14^. Lastly, the model trained after PLHM, which showed the best generalisation ability, obtained medium performance on the validation set signalling a reduction in overfitting. The most important features for this model were predominantly features from the MYO, which made sense for the classification task at hand, since HCM is most evident when looking at the myocardium.

This work presents several limitations. First, the dataset was not perfectly balanced across the five centres and the population was not controlled by age, sex, body size, or myocardial volume, which could result in dissimilarities across feature distributions. However, no significant differences were found under a Mann–Whitney *U* test in the volumes of the different ROIs between centres.

The choice of HCM classification as a metric for generalisation has some drawbacks, since the heart suffers morphological changes and some texture features are known to be correlated with the shape^16^. This could contribute to overestimating the generalisation ability. The inclusion of other pathologies that greatly affect the myocardium, such as myocarditis or infarction, would potentially result in a less biased generalisation loss estimation.

Finally, according to the Imaging Biomarker Standardization Initiative, ISBI, second order features from different texture matrices may be modelled better with different intensity discretisation levels (e.g. GLSZM are better characterised for low discretisation levels while it is the opposite for GLCM)^17^. In this work, the same discretisation level was used for all features.

## Conclusions

In summary, this study showed that centre-related information removal does not imply good generalisation ability for classification. ComBat harmonisation was able to remove centre-related information from radiomic features satisfactorily, while showing limited generalisation ability. PLHM normalisation resulted in the best generalisable model for classification of healthy subjects from HCM patients. The choice of reference template when performing histogram matching may affect the results. PLHM was robust against a change of reference population. Finally, the radiomic features from GLSZM, GLDM and GLRLM families showed greater variability than first order and GLCM features. Further studies with a larger sample size are needed in order to replicate the results presented and to assess the effect of different biological covariates.

## Material and methods

### Data and feature extraction

A subset of 218 cardiac magnetic resonance studies from the Multi-Centre, Multi-Vendor and Multi-Disease Cardiac Image Segmentation Challenge (M &Ms) dataset was considered^18^. In particular, healthy subjects as well as patients with hypertrophic cardiomyopathy (HCM) were selected from the five available centres. The exact distribution across the five centres is presented in Table 2. All the scanners considered had a field strength of 1.5T and the averaged in-plane resolution ranged from 0.85 to 1.45 mm. More detailed information about the scanners used can be found in Table 3.

**Table 2:** Distribution of diseases per centre considered in the analysis.

Centre	Creu Blanca	Dexeus	Sagrada Familia	Universitätsklinikum Hamburg-Eppendorf	Vall d’Hebron	Total
	Canon	General Electric	Philips	Philips	Siemens	
Healthy	14	11	33	32	22	112
HCM	15	5	37	14	25	106

**Table 3:** Average specifications for the studies acquired in the five different centres.

Centre	Vendor	Model	In-plane resolution (mm)	Slice thickness (mm)	Number of slices	Intensities range
Vall d’Hebron	Siemens	Magnetom Avanto	1.32	9.2	12	0–1193
Sagrada Familia	Philips	Achieva	1.20	9.9	10	0–357
Universitätsklinikum Hamburg-Eppendorf	Philips	Achieva	1.45	9.9	11	0–3725
Dexeus	General Electric	Signa Excite	1.36	10	12	0–3030
Creu Blanca	Canon	Vantage Orian	0.85	10	13	0–14,442

Each study consisted of a short-axis cine cardiac magnetic resonance volume. Segmentations of three anatomical ROIs, the left and right ventricle cavities (LV and RV, respectively) and the left ventricle myocardium (MYO), were provided for two temporal phases, ES and ED. The delineations were revised to follow the same Standard Operating Procedure to avoid the introduction of further bias due to inter-observer variability.

Radiomic features were extracted using the PyRadiomics library^19^, version 3.0.1. Prior to the extraction, all images were resized to match the same spatial resolution of $1\times 1$ mm$^2$, since radiomic features have been shown to intrinsically depend on voxel size and on the number of voxels^16^. Fixed bin widths of 25 and 0.05 were used during feature extraction for images before and after normalisation, respectively. This resulted in a good balance between number of bins and computing requirements. The number of bins after normalisation ranged between 20 and 80, depending on the intensity values for each ROI. Only images without normalisation gave a large variability in terms of number of bins (from 14 to 570).

A total of 100 features were extracted per ROI. They include shape features and first and second order texture features. In this work, only texture features were used, since shape depends only on the ROI segmentation and not on the image intensity. First order texture features refer to commonly used statistical metrics to describe the histogram of intensity values such as mean, minimum, maximum, kurtosis, skewness, entropy and energy, among others. Second order texture features are statistical measures extracted from the four texture matrices considered in this library: Gray Level Co-occurrence Matrix (GLCM), Gray Level Size Zone Matrix (GLSZM), Gray Level Run Length Matrix (GLRLM) and Gray Level Dependence Matrix (GLDM). These features account for different details in the spatial coarseness, variability, heterogeneity and symmetry of textures. A complete list of the features considered is included in the supplementary material.

### Normalisation techniques

Four normalisation techniques were considered at the image level:

- R: image intensity rescaling to the range 0–1,
- N: image intensity normalisation (mean subtraction and division by the standard deviation),
- HM: histogram matching using scikit-image^20^, version 0.17.2,
- PLHM: piecewise linear histogram matching^21^, also referred to as Nyúl–Udupa normalisation.

For the histogram matching transformation, an image intensity histogram is interpolated so that it matches a template histogram. In this work, a subject was selected visually from Sagrada Familia as the template after ensuring that the image did not present artifacts. For the PLHM transformation, the code implementation by Reinhold et al.^22^ was employed. In this case, a batch of images from one centre was needed to obtain the averaged histogram deciles (landmarks) that were then used as reference for the transformation of new image histograms. The landmarks were computed for studies from Sagrada Familia. All transformations were applied both to the whole image and at ROI level, independently. Data from Sagrada Familia were used as reference, since it was the centre with the greatest number of scans.

Regarding feature-based normalisation techniques, the empirical Bayes harmonisation method proposed by Johnson and Rabinovic (ComBat) was considered^13,23^. This method assumes that the contributions to the final feature values can be separated in biological covariates (e.g., pathology) and centre effects (e.g., different scan manufacturers). Then, the empirical Bayes method is used to estimate the distributions for these terms from original data and adjust the final feature values to remove centre effects. The ComBat method is robust against outliers and does not need large sample sizes for each centre batch, which makes it a good option for the current study. However, feature distributions are assumed to follow normal distributions for each centre separately, a requirement not always satisfied by the data. For this reason, a quantile transformation (scikit-learn^24^, version 0.23.2) had to be applied to all radiomic features for each institution independently before ComBat could be used (we used 20 as number of quantiles). The Python implementation of ComBat by Fortin et al.^23^, available at github.com/Jfortin1/ComBatHarmonization, was used. Five batches were used during the harmonisation process, one for each centre. A parametric adjustment was chosen for fitting the batch effect parameters^13^, and the alignment was performed over a virtual reference frame instead of over one of the five batches. No covariates were used along with ComBat harmonisation.

### Variability assessment

Radiomics variability across centres was assessed by computing the Jensen–Shannon divergence (JSD) between pairs of feature distributions obtained for healthy subjects within the different ROIs. The HCM pathological group was not considered in this analysis since the possible existence of different HCM sub-groups could introduce uncontrolled bias to the results. Moreover, in order to avoid redundancy of features in the results, a prior sequential feature selection step was conducted to remove features that showed a square cross correlation coefficient greater or equal than 0.9 with any previous feature following the ordering provided by PyRadiomics (see Table S1 in the supplementary material). The JSD gives a positive measure of how similar two distributions are, with 0 the value obtained when the two distributions are identical. A threshold of 0.01 JSD was selected based on the median of the overall distribution as the relative point where changes in feature proportions were to be assessed.

Then, in order to analyse model generalisation, two tasks were proposed. First, the amount of centre-encoded information after the application of each normalisation technique was measured by training Random Forest (RF) models to identify the source centre for each feature set. The hypothesis was that features with less information about their centre of origin should be more difficult to differentiate and thus, more similar between centres, enhancing the generalisation. Secondly, model generalisation was assessed directly by training RF models for patient classification into healthy or HCM groups, for each normalisation technique. RF were chosen over other techniques due to their simplicity to train and their effectiveness to model non-linear relations between input and output. For all cases, a random seed was fixed before training each model to make the results reproducible.

For the centre identification task, models were trained either with first order or second order features as input variables for each ROI separately (LV, MYO and RV). A five-fold cross-validation was used for obtaining an estimate of the average classification accuracy with reduced bias. Notably, in this case, a lower accuracy represents that features carry less centre information.

For the patient classification task, models were trained with a combination of first and second order features from all three ROIs, so that RF was able to select the most predictive features during training. Five runs of the same cross-validation scheme were considered in this case (five different random seeds), since the variability in models was higher and the accuracy estimates showed greater bias. In particular, these models were trained with features from only one dataset (Vall d’Hebron ($n=38$) or Sagrada Familia $n=56$, as these centres had a greater number of samples) and tested on the other four. No feature selection was conducted prior to model training. The most important features for the best performing models were obtained with the mean impurity decrease method^15^, also called Gini importance. For these models, a greater accuracy represents better generalisation. All models were assessed with balanced accuracy, given the imbalanced nature of the dataset.

### Ethics

All patients signed the informed consent, the study protocol was approved by the Ethical Committee for Clinical Research for each institution involved, and it follows the ethical guidelines of the Declaration of Helsinki.

## Supplementary Information

Supplementary Information.

## Data Availability

The datasets analysed in this study can be found openly at the M &Ms Challenge webpage: ub.edu/mnms.
