# Supplementary material for: Minimising multi-centre radiomics variability through image normalisation: a pilot study
Source: Sci Rep. 2022 Jul 22;12:12532. doi: 10.1038/s41598-022-16375-0 (PMC9307565; doi:10.1038/s41598-022-16375-0)
Supplement: Supplementary file 1 — Supplementary Information. [file 41598_2022_16375_MOESM1_ESM.pdf]

Supplementary material

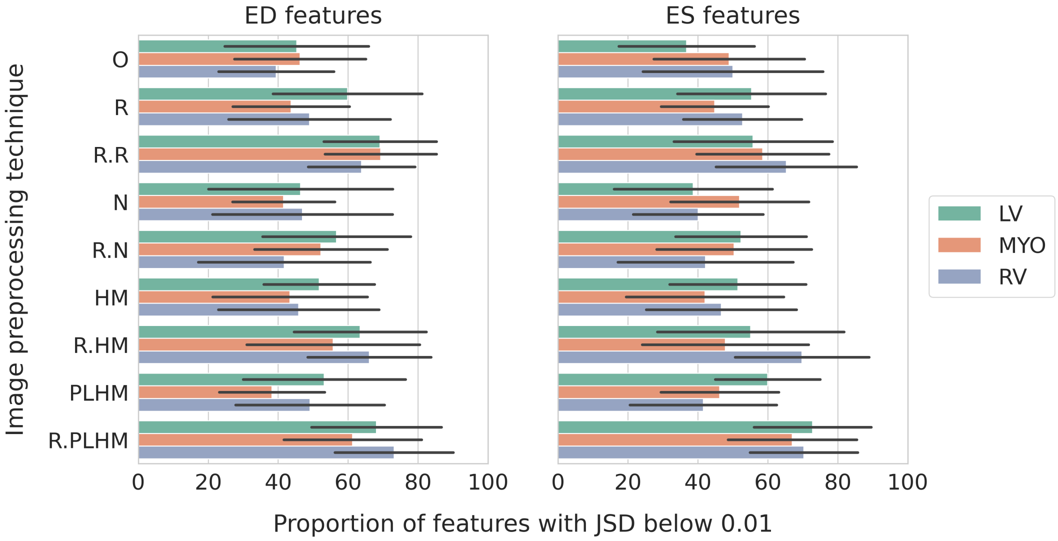

Figure S1. Percentage of texture features below the 0.01 JSD threshold for each ROI for healthy subjects. Results are averaged over feature types and centre pairs and separated in ED and ES frames. Only features with square cross-correlation below 0.9 were considered. The black lines represent the standard deviation. O: original images (without normalisation), R: image intensity recaling, N: image intensity normalisation, HM: histogram matching and PLHM: piecewise linear histogram matching. An "R." in front of a method means that it is applied at ROI level.

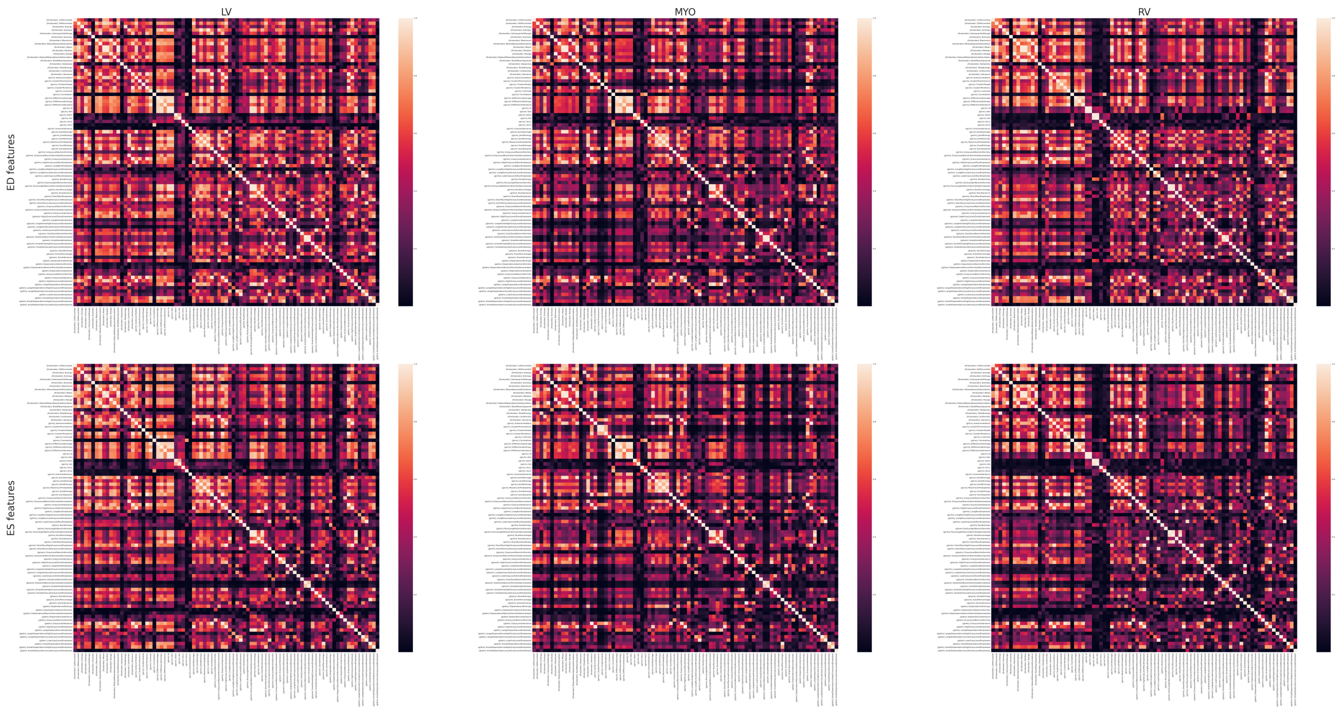

Figure S2. Pairwise square correlation for features extracted from the three different ROIs without the application of any normalisation technique. The correlation between features was very similar for the different preprocessing techniques, showing a negligible standard deviation. Zoom in to see in more detail.

Table S1. List of radiomic features extracted with PyRadiomics. We refer the reader to the library documentation (https://pyradiomics.readthedocs.io/) for the specific definition and interpretation of each feature.

Family	Index	Feature
Shape	1	Elongation
	2	Flatness
	3	LeastAxisLength
	4	MajorAxisLength
	5	Maximum2DDiameterColumn
	6	Maximum2DDiameterRow
	7	Maximum2DDiameterSlice
	8	Maximum3DDiameter
	9	MeshVolume
	10	MinorAxisLength
	11	Sphericity
	12	SurfaceArea
	13	SurfaceVolumeRatio
	14	VoxelVolume
First order	15	10Percentile
	16	90Percentile
	17	Energy
	18	Entropy
	19	InterquartileRange
	20	Kurtosis
	21	Maximum
	22	MeanAbsoluteDeviation
	23	Mean
	24	Median
	25	Minimum
	26	Range
	27	RobustMeanAbsoluteDeviation
	28	RootMeanSquared
	29	Skewness
	30	TotalEnergy
	31	Uniformity
	32	Variance
GLCM	33	Autocorrelation
	34	JointAverage
	35	ClusterProminence
	36	ClusterShade
	37	ClusterTendency
	38	Contrast
	39	Correlation
	40	DifferenceAverage
	41	DifferenceEntropy
	42	DifferenceVariance
	43	JointEnergy
	44	JointEntropy
	45	Imc1
	46	Imc2
	47	Idm
	48	Idmn
	49	Id
	50	Idn
GLCM	51	InverseVariance
	52	MaximumProbability
	53	SumEntropy
	54	SumSquares
GLRLM	55	GrayLevelNonUniformity
	56	GrayLevelNonUniformityNormalized
	57	GrayLevelVariance
	58	HighGrayLevelRunEmphasis
	59	LongRunEmphasis
	60	LongRunHighGrayLevelEmphasis
	61	LongRunLowGrayLevelEmphasis
	62	LowGrayLevelRunEmphasis
	63	RunEntropy
	64	RunLengthNonUniformity
	65	RunLengthNonUniformityNormalized
	66	RunPercentage
	67	RunVariance
	68	ShortRunEmphasis
	69	ShortRunHighGrayLevelEmphasis
	70	ShortRunLowGrayLevelEmphasis
GLSZM	71	GrayLevelNonUniformity
	72	GrayLevelNonUniformityNormalized
	73	GrayLevelVariance
	74	HighGrayLevelZoneEmphasis
	75	LargeAreaEmphasis
	76	LargeAreaHighGrayLevelEmphasis
	77	LargeAreaLowGrayLevelEmphasis
	78	LowGrayLevelZoneEmphasis
	79	SizeZoneNonUniformity
	80	SizeZoneNonUniformityNormalized
	81	SmallAreaEmphasis
	82	SmallAreaHighGrayLevelEmphasis
	83	SmallAreaLowGrayLevelEmphasis
	84	ZoneEntropy
	85	ZonePercentage
	86	ZoneVariance
GLDM	87	DependenceEntropy
	88	DependenceNonUniformity
	89	DependenceNonUniformityNormalized
	90	DependenceVariance
	91	GrayLevelNonUniformity
	92	GrayLevelVariance
	93	HighGrayLevelEmphasis
	94	LargeDependenceEmphasis
	95	LargeDependenceHighGrayLevelEmphasis
	96	LargeDependenceLowGrayLevelEmphasis
	97	LowGrayLevelEmphasis
	98	SmallDependenceEmphasis
	99	SmallDependenceHighGrayLevelEmphasis
	100	SmallDependenceLowGrayLevelEmphasis

GLCM: Gray Level Co-ocurrence Matrix, GLRLM: Gray Level Run Length Matrix, GLSZM: Gray Level Size Zone Matrix and GLDM: Gray Level Dependence Matrix.

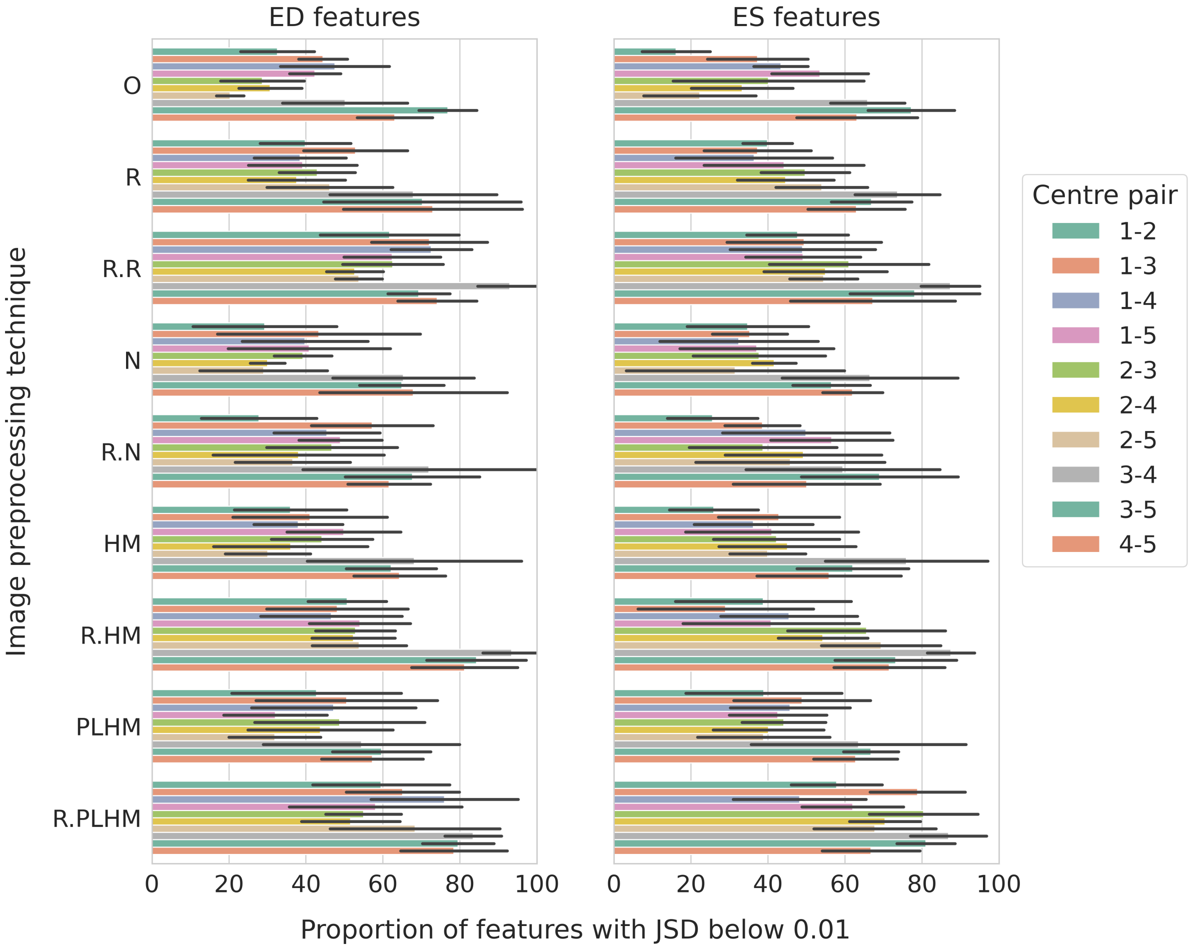

Figure S3. Percentage of texture features below the 0.01 JSD threshold for each centre pair, indexed in alphabetical order as in Table 2, for healthy subjects. Results are averaged over feature types and ROIs and separated in ED and ES frames. Only features with square cross-correlation below 0.9 were considered. The black lines represent the standard deviation. O: original images (without normalisation), R: image intensity recaling, N: image intensity normalisation, HM: histogram matching and PLHM: piecewise linear histogram matching. An "R." in front of a method means that it is applied at ROI level.

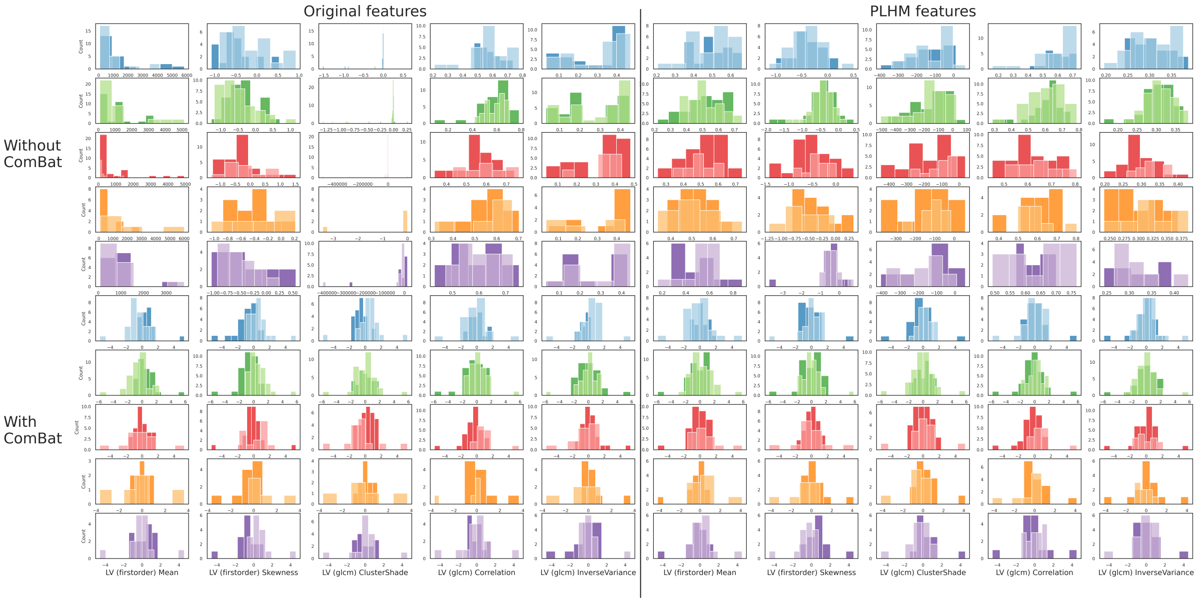

Figure S4. Comparison of histograms of five different radiomic features from the LV across centres and for different normalisation methods. Histograms and separated for healthy (brighter color) and HCM subjects (lighter color). Centres are presented in different colors and different rows following the ordering in Table 3. The first five rows correspond for methods without ComBat harmonisation, while the last five rows represent the same features with ComBat harmonisation. The first five columns are distributions of features extracted from original images, while the last five are features extracted after PLHM.

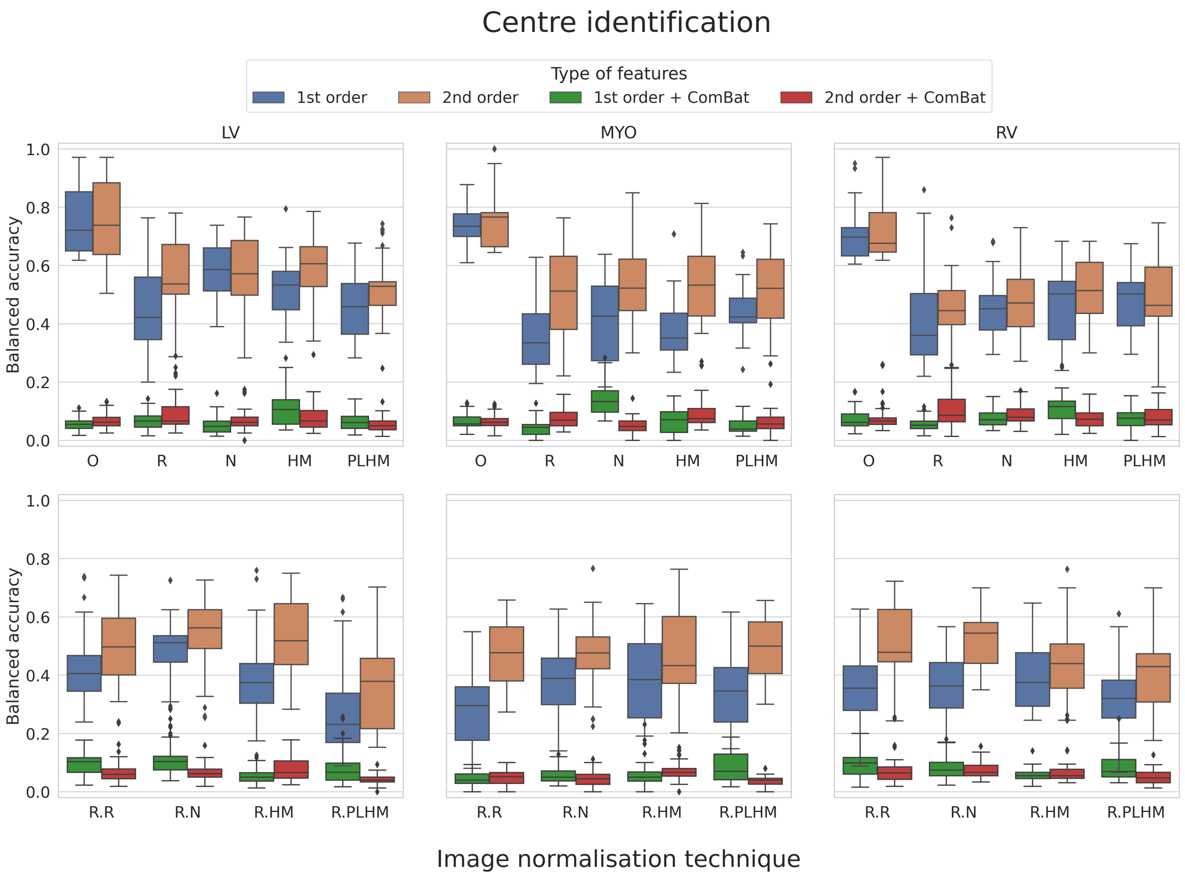

Figure S5. Balanced accuracy of random forest models when predicting the centre of origin of HCM subjects for first and second order texture features before and after the application of ComBat harmonisation. The row above corresponds to image preprocessing techniques applied at the whole image level, while in the row below they are applied at the ROI level. O: original images (without normalisation), R: image intensity recaling, N: image intensity normalisation, HM: histogram matching and PLHM: piecewise linear histogram matching. An “R.” in front of a method means that it is applied at ROI level.

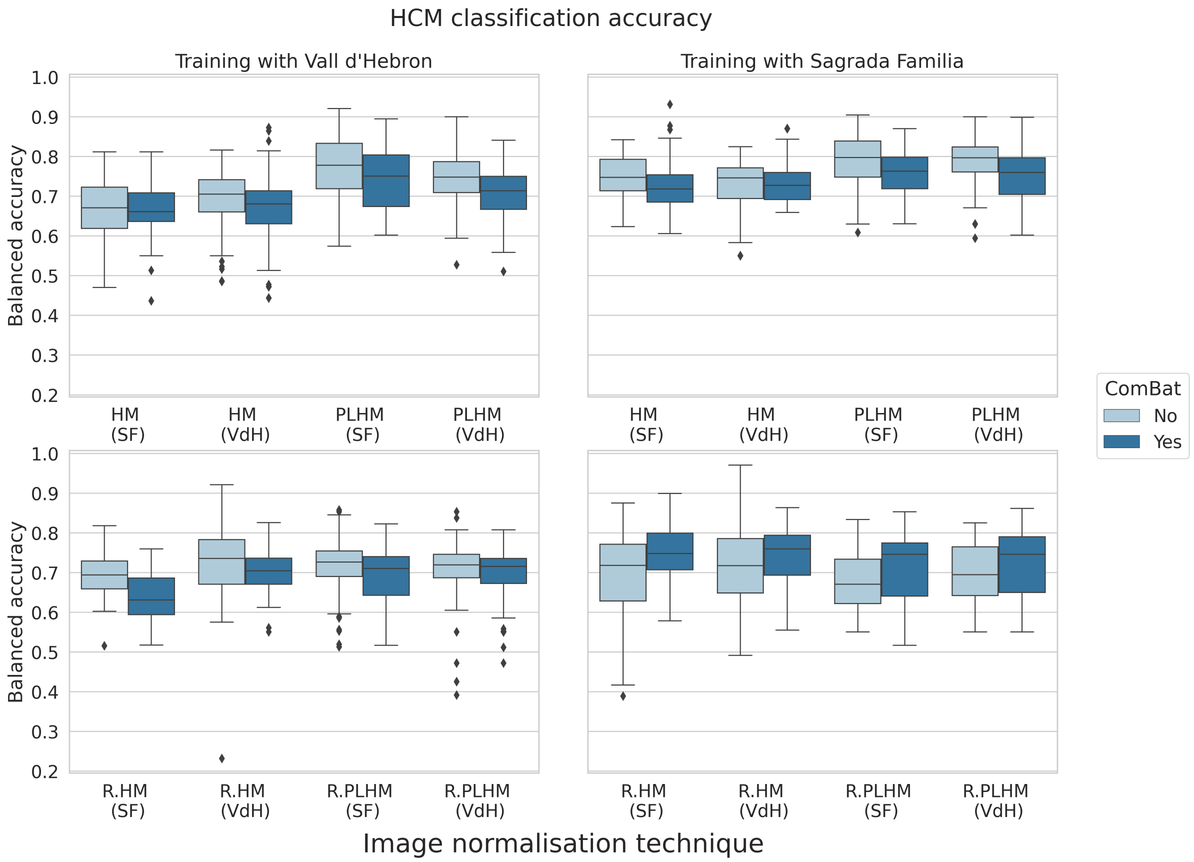

Figure S6. Comparison of balanced accuracy for models trained with two different reference templates from Vall d'Hebron (VdH) and Sagrada Familia (SF) on the HCM classification task. All models were trained with a combination of first and second order texture features from all ROIs. The first column corresponds to models trained with features extracted from Vall d'Hebron studies, while models in the second column were trained with features from Sagrada Familia studies. The row above corresponds to image preprocessing techniques applied at the whole image level, while in the row below they are applied at the ROI level. HCM: Hypertrophic cardiomyopathy, VdH: Vall d'Hebron, SF: Sagrada Familia, HM: histogram matching and PLHM: piecewise linear histogram matching. An "R." in front of a method means that it is applied at ROI level.

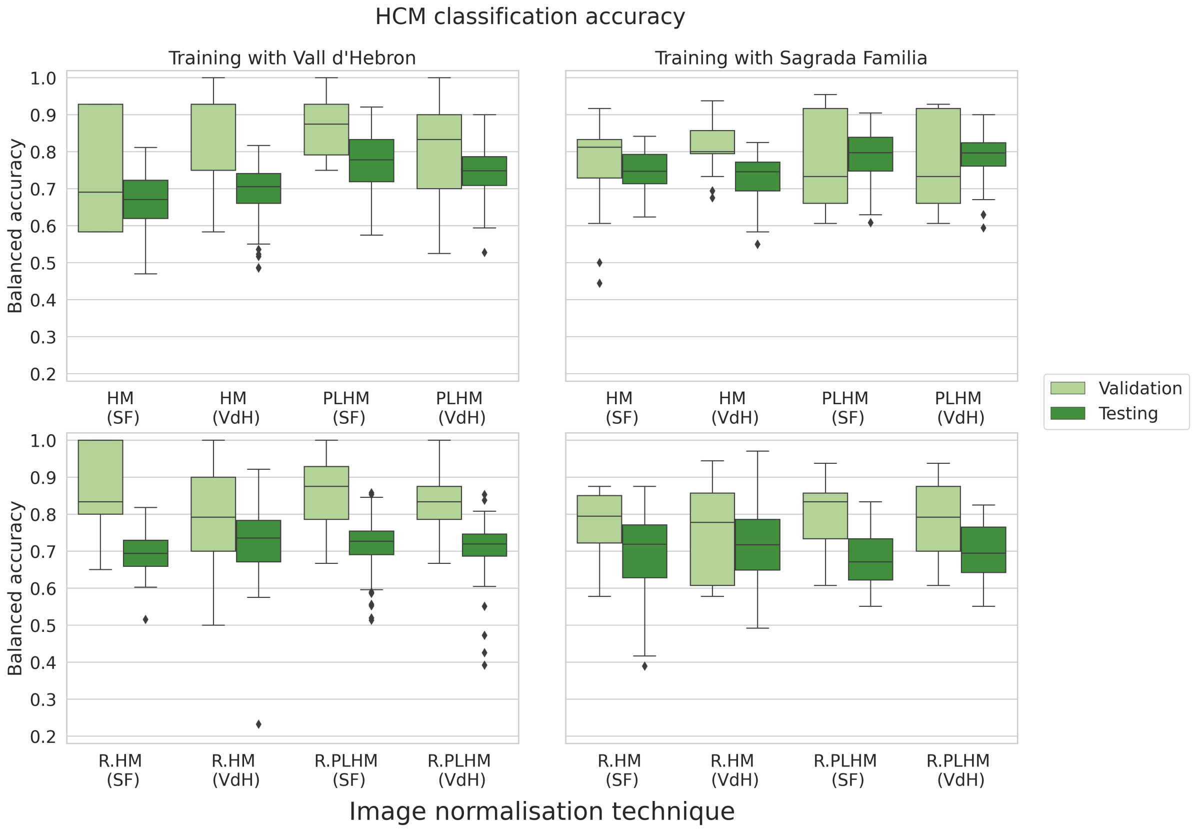

Figure S7. Comparison of validation (same domain) and testing (unseen centres) balanced accuracy for models trained with two different reference templates from Vall d'Hebron (VdH) and Sagrada Familia (SF) on the HCM classification task. Results are presented without ComBat harmonisation. All models were trained with a combination of first and second order texture features from all ROIs. The first column corresponds to models trained with features extracted from Vall d'Hebron studies, while models in the second column were trained with features from Sagrada Familia studies. The row above corresponds to image preprocessing techniques applied at the whole image level, while in the row below they are applied at the ROI level. HCM: Hypertrophic cardiomyopathy, O: original images (without normalisation), R: image intensity recaling, N: image intensity normalisation, HM: histogram matching and PLHM: piecewise linear histogram matching. An "R." in front of a method means that it is applied at ROI level.
